# Platelet Transfusion Practices and Outcomes in Neonates and Children

**DOI:** 10.1001/jamanetworkopen.2025.54531

**Published:** 2026-01-28

**Authors:** Ruchika Goel, Oliver Karam, Donald E. Warden, Rebecca J. Birch, Thomas C. Binns, Rowena Punzalan, Nareg Roubinian, Nalan Yurtsever, Ravi M. Patel, Daniel Bougie, Naomi Luban, Cassandra D. Josephson, Martha Sola-Visner, Marianne E. Nellis

**Affiliations:** 1 Department of Pathology, Johns Hopkins University, Baltimore, Maryland; 2 Division of Hematology and Oncology, Simmons Cancer Institute at SIU School of Medicine, Springfield, Illinois; 3 Pediatric Critical Care Medicine, Department of Pediatrics, Yale, New Haven, Connecticut; 4 Westat, Rockville, Maryland; 5 Department of Laboratory Medicine, Yale University, New Haven, Connecticut; 6 Versiti Blood Research Institute, Milwaukee, Wisconsin; 7 Kaiser Permanente Northern California Division of Research, Pleasanton, California; 8 Vitalant Research Institute, San Francisco, California; 9 Department of Neonatology, Emory University and Children's Healthcare of Atlanta, Atlanta, Georgia; 10 Thrombosis and Hemostasis Program, Versiti Blood Research Institute, Milwaukee, Wisconsin; 11 Children’s National Medical Center, Washington, DC; 12 Johns Hopkins All Children’s Hospital, St Petersburg, Florida; 13 Division of Newborn Medicine, Boston Children’s Hospital and Harvard Medical School, Boston, Massachusetts; 14 Division of Critical Care Medicine, Department of Pediatrics, Weill Cornell Medicine, New York, New York

## Abstract

**Importance:**

Platelet transfusions are critical interventions for neonates and children who are at risk of or who are experiencing bleeding.

**Objective:**

To describe the epidemiology of platelet transfusions and associations of blood donor and platelet characteristics with posttransfusion platelet increments, transfusion burden, and clinical outcomes in neonates and children.

**Design, Setting, and Participants:**

This cohort study included patients younger than 18 years of age (with birth weights higher than 2500 g) between April 1, 2019, and June 30, 2023, from the Recipient Epidemiology and Donor Evaluation Study-IV-Pediatric Vein-to-Vein multicenter, retrospective, donor-component-recipient−linked database. Neonates were considered individuals less than 28 days of age; otherwise, participants were considered older children.

**Exposure:**

Platelet transfusion.

**Main Outcome and Measures:**

The main outcomes were posttransfusion platelet increments and subsequent transfusion burden associated with platelet processing and donor characteristics.

**Results:**

Of 249 340 inpatient encounters, platelet transfusion was reported in 8874 (3.6%) patients (4934 of 131 592 encounters [3.7%] male; median [IQR] age 2.5 [0.6-11.2] years). Platelet transfusion was lowest among children younger than 1 year of age (2.6%) and highest among children 1 to less than 6 years of age (4.7%; *P* < .001). The median (IQR) dose was 14.9 mL/kg for neonates and 9.6 mL/kg for older children. After excluding patients with bleeding, most transfusions in neonates (67.8%) and older children (81.0%) were performed at pretransfusion platelet counts greater than 25 × 10^3^/µL and greater than 10 × 10^3^/µL, respectively. Median (IQR) pretransfusion platelet counts in neonates (34 × 10^3^/µL [20-54 × 10^3^/µL]) were significantly higher than in older children (22 × 10^3^/µL [11-40 × 10^3^/µL]; *P* < .001). Pathogen reduction (PR) (adjusted odds ratio [AOR], 0.82 [95% CI, 0.73-0.92]), use of platelet additive solution (PAS) (AOR, 0.32 [95% CI, 0.27-0.37]), platelet storage duration longer than 3 days (AORs ranged from 0.67 [95% CI, 0.58-0.76] to 0.82 [95% CI, 0.76-0.88]), male sex (AOR, 0.92 [95% CI, 0.86-0.98]), and donor age 40 years or older (AOR, 0.79 [95% CI, 0.72-0.86]) were associated with lower platelet increments (all *P* < .001). Use of PR platelets (adjusted rate ratio [ARR], 1.05 [95% CI, 1.02-1.07), use of PAS (ARR, 1.44 [95% CI, 1.40-1.47]), storage duration longer than 3 days (ARR, 1.11 [95% CI, 1.09-1.13] for 4 to <5 days and ARR, 1.28 [95% CI, 1.26-1.30] for ≥5 days), and donor age of 40 years or older (ARR, 1.15 [95% CI, 1.13-1.17] for 40 to <60 years and ARR, 1.10 [95% CI, 1.08-1.12] for ≥60 years) on the first transfusion were associated with a significantly higher rate of receiving a subsequent transfusion. These factors were not associated with hospital length of stay or mortality.

**Conclusions and Relevance:**

In this multicenter, donor-product-recipient linked cohort study, after excluding patients with bleeding, most transfusions among neonates and older children were at high pretransfusion platelet counts. Use of PAS, PR, longer storage duration, male donors, and donor age older than 40 years were independently associated with lower posttransfusion platelet increments. Use of PAS, PR, platelet storage longer than 3 days, and donor age 40 years or older were associated with a significantly higher overall platelet transfusion burden but were not associated with hospital length of stay or mortality. These results have important implications for transfusion practices for platelets among neonates and children and need validation in well-designed prospective studies.

## Key Points

**Question:**

What is the epidemiology of platelet transfusions in pediatric and neonatal recipients, and are donor and platelet processing factors associated with posttransfusion platelet increments, overall transfusion burden, and clinical outcomes?

**Findings:**

In this cohort study involving 249 340 inpatient encounters, 3.6% of patients received platelet transfusions. Lower platelet increments and a higher transfusion burden were observed for the use of platelet additive solution, pathogen reduction, and platelets with longer storage durations, and there was a similar association with donor age; whereas these donor and platelet factors were not associated with adverse clinical outcomes.

**Meaning:**

This study describes platelet transfusion practices in neonates and children and various platelet processing and donor characteristics associated with posttransfusion platelet increments and overall transfusion burden.

## Teaser

This cohort study describes platelet transfusion practices and assesses the associations of blood donor and platelet characteristics with posttransfusion platelet increments, transfusion burden, and clinical outcomes among neonates and older children.

## Introduction

Platelet transfusions are a critical intervention for neonates and children who are either bleeding (therapeutic transfusions) or at risk of bleeding (prophylactic transfusions).^1,2,3,4,5,6,7^ Platelet donor characteristics, such as sex and age, and further processing and storage of platelet units may affect platelet increment (PI), overall transfusion burden, and clinical outcomes in platelet recipients. In adult recipients, donor sex and age affect platelet metabolism at the time of donation and following storage, resulting in lower posttransfusion PI.^8^ In certain adult cohorts, platelets of older storage age have been independently associated with adverse outcomes, including suboptimal PI, increased nonhemolytic transfusion reactions, and adverse pulmonary events, such as transfusion-associated dyspnea and transfusion-related acute lung injury.^9,10,11^ In neonates, results from retrospective data suggest that processing practices such as pathogen reduction (PR), storage length, and ABO matching may affect the posttransfusion platelet count, but little is known about clinical outcomes in these patients.^12,13^ This information is crucial because identifying the influence of donor characteristics and platelet processing factors on transfusion effectiveness and clinical outcomes can inform better transfusion practices.

Platelet collection and processing steps are highly variable among blood centers. Platelets for transfusion can be derived from a whole blood donation or can be collected by apheresis, which returns all other blood components to the donor. In the blood bank, platelets for pediatric use are sometimes irradiated to prevent graft-vs-host disease. Platelets can also undergo PR, consisting of treatment with an agent that on exposure to UV light cross-links nucleic acid strands and impairs the replication of pathogens and leukocytes, thus preventing transfusion-transmitted infections and graft-vs-host disease. Platelets can be stored or suspended in either pure autologous plasma or an electrolyte solution (platelet additive solution [PAS]) that replaces approximately 70% of the plasma, and sampled for bacterial detection, allows for up to a 7-day shelf-life.

Pediatric and neonatal studies have suggested substantial potential adverse events associated with platelet transfusions,^14,15,16,17^ including increased incidence of thrombosis, lung injury, mortality rate, poor neurodevelopmental outcomes, and paradoxically increased risk of bleeding.^16,18^ To mitigate the morbidity associated with platelet transfusions, it is imperative to investigate the characteristics of platelet transfusions from donation through the processing steps and the subsequent effect of platelet transfusions on clinical outcomes for the recipients by using a vein-to-vein analysis.^4^ The present study used a donor-component-recipient–linked database to (1) describe the epidemiology of platelet transfusions, (2) assess the posttransfusion platelet count increment, and (3) explore the associations of donor characteristics and platelet processing steps with transfusion burden, overall transfusion effectiveness, and clinical outcomes among both pediatric and neonatal recipients.

## Methods

### Data Source

The Recipient Epidemiology and Donor Evaluation Study-IV-Pediatric (REDS-IV-P) program, a 7-year program funded by the National Heart, Lung and Blood Institute, established a Vein-to-Vein database that includes comprehensive donor, component, and recipient records for patients with or without transfusions.^19,20^ Data were collected from April 1, 2019, to March, 31, 2024, across 4 geographic locations in the US, including 7 blood centers and 22 hospitals, of which 6 were children’s hospitals.^2^ The Westat institutional review board determined that the study met the 45 CFR 46.104(d) category(4iii) for exempt from review with a waiver of informed consent. This observational cohort study was designed and reported in accordance with the Strengthening the Reporting of Observational Studies in Epidemiology (STROBE) guideline for cohort studies.

### Epidemiology of Platelet Transfusions

Inpatient encounters for patients aged 0 to younger than 18 years were included (excluding individuals with birth weights <2500 g). Neonates were considered individuals less than 28 days of age; otherwise, participants were considered older children. For multiple admissions, each admission was considered a unique encounter. A platelet transfusion event was defined as issue of a platelet product by the transfusion service within the encounter start and end times. Transfusion incidence was calculated as a binomial proportion of encounters during which at least 1 platelet product was issued. Transfusion incidence was calculated for the total population and by patient sex, age, and self-reported race and ethnicity at encounter. Race and ethnicity were assessed to characterize the cohort and assess associations with transfusion outcomes. Categories included Asian, Black, Native American or American Indian, Native Hawaiian or Pacific Islander, White, multiracial, missing, or unknown.

Characteristics of the platelet products transfused were described by percentages (the number with the characteristic divided by the total platelet products transfused, multiplied by 100). Concomitant red blood cell, plasma, or cryoprecipitate transfusion was defined as the respective transfusion within 6 hours of the platelet transfusion. The Information Standard for Blood and Transplant (ISBT) code was used to determine the collection method (apheresis-derived or whole blood–derived), storage in PAS (yes or no), PR status (yes or no), and irradiated status (yes or no) of the platelet products. Product issue date and time, donation identification number, ISBT code, and the aliquot code (if applicable) were used to link the transfused products to the donor or donation and blood center and hospital transfusion service data. These data provided the donor demographics (sex, age, race, ethnicity, donor ABO, and donation dates). Storage duration was calculated as the difference in days between the transfusion and donation date and time. For cases in which the transfusion start was unknown (2.0% of events), the issue date and time was used.

### Assessment of Posttransfusion Platelet Count Increment

The unit of analysis was the platelet transfusion. Platelet transfusions without a pretransfusion platelet count measured within 24 hours prior (5.7%) and without a posttransfusion platelet count measured within 24 hours after (4.9%) were excluded from the analysis. Patients receiving extracorporeal membrane oxygenation, undergoing cardiopulmonary bypass surgery, or suspected of bleeding—based on a definition using specific clinical and laboratory criteria (eAppendix in Supplement 1)—were excluded from the increment analysis (n = 9212 [22.6%]) (eFigure 1and eTable 1 in Supplement 1).

### Associations of Platelet Concentrate Factors and Donor Characteristics With Transfusion Burden and Clinical Outcomes

The unit of analysis was the encounter. Encounters with patients receiving extracorporeal membrane oxygenation, undergoing cardiopulmonary bypass surgery, or suspected of bleeding (n = 3055 [34.4%]) were excluded from the analysis, as were encounters with no pretransfusion platelet count measures (n = 1218 [13.7%]) (eFigure 1 and eTable 1 in Supplement 1).

Transfusion burden was defined as the number of platelet transfusions during the encounter, length of hospital stay was discretized into a binary variable representing discharge before or after 1 week (7 days), and in-hospital mortality was defined as any encounter resulting in in-hospital death. For the clinical outcomes (length of hospital stay and in-hospital mortality), we considered the platelet and donor characteristics of the first transfusion that occurred during the encounter.

### Statistical Analysis

PI was calculated as the difference between the posttransfusion and pretransfusion platelet counts. To assess whether the pretransfusion platelet count was associated with the PI, density plots of the PI and reported median and IQR were stratified by categorial pretransfusion platelet counts. We estimated the risk ratio of the length of hospital stay and the rate ratios of transfusion burden and mortality using Poisson regression with robust error variance and a log link using the SAS procedure GENMOD. Analyses of transfusion burden and mortality were adjusted for an offset of length of hospital stay to account for varying exposure time and to generate rate ratios.

For all models (including PI >15 × 10^3^/µL), missing data for donor sex (values coded as unknown, missing, or declined) were grouped and analyzed together as a distinct category. For other variables with missing data (donor age and platelet storage duration), analyses were completed on a complete-case basis without imputation. eTable 1 in Supplement 1 presents the population characteristics between patients included in the models and those excluded. To assess the association between platelet processing factors and platelet donor factors with the PI, the PI was dichotomized as 15 × 10^3^/µL or less vs higher than 15 × 10^3^/µL (to convert to SI units [× 10^9^/L], multiply by 1.0). We estimated the odds ratio (OR) of the dichotomized PI (odds of PI >15 × 10^3^/µL) for platelet concentrate factors (PR, PAS, and storage duration) and donor characteristics (sex and age) using mixed-effects logistic regression, adjusting for random effects of repeated measures within subjects (SAS procedure GLIMMIX). The model of donor characteristics was also adjusted for random effects of repeated measures within donors. Statistical significance was determined at a 2-sided α of .05. Analyses were conducted with SAS, version 9.4 TS1M8 (SAS Institute Inc) and R version, 4.4. 1 (R Project for Statistical Computing).

## Results

### Population

A total of 249 340 inpatient neonatal and pediatric encounters were analyzed (Table 1). At least 1 platelet transfusion was reported in 8874 (3.6%) of all patients (female, 3939 of 117 731 encounters [3.3%]; male, 4934 of 131 592 encounters [3.7%]), with a median (IQR) age of 2.5 (0.6-11.2) years and median (IQR) weight of 16.6 (7.3-42.7) kg. Children younger than 1 year of age had the lowest percentage platelet transfusion (2.6%), whereas children 1 to less than 6 years of age had the highest (4.7%; *P* < .001). Black race had the lowest percentage of platelet transfusion (2.3%), compared with Asian (4.2%), Native American or American Indian (5.4%), Native Hawaiian or Pacific Islander (3.8%), White (4.2%), multiracial (4.0%), and unknown (8.2%) races (*P* < .001).

**Table 1.** Inpatient Neonatal and Pediatric Encounters Reporting Platelet Transfusions

Encounter characteristic	No. of inpatient encounters reporting platelet transfusions/total No. of inpatient encounters (transfusion incidence %)
Total inpatient neonatal and pediatric encounters	8874/249 340 (3.6)
Sex	
Female	3939/117 731 (3.3)
Male	4934/131 592 (3.7)
Weight, median (IQR), kg^a^	16.6 (7.3-42.7)
Age, median (IQR), y	2.5 (0.6-11.2)
Age group, y	
<1	2610/100 886 (2.6)
1 to <6	2607/55 011 (4.7)
6 to <13	1999/45 058 (4.4)
13 to <18	1658/48 385 (3.4)
Race and ethnicity	
Asian	582/13 999 (4.2)
Black	862/36 954 (2.3)
Multiracial	59/1476 (4.0)
Native American or American Indian	49/911 (5.4)
Native Hawaiian or Pacific Islander	1467/38 251 (3.8)
White	582/13 999 (4.2)
Unknown	5740/152 744 (8.2)

^a^ Missing data for patient weight 12.9%.

A total of 40 779 platelet transfusion episodes were analyzed (eTable 2 in Supplement 1). The median (IQR) number of platelet transfusions per patient encounter was 2 (1-4). At least 1 concomitant red blood cells, plasma, or cryoprecipitate transfusion (within 6 hours of a platelet transfusion) was reported in 32.5% of cases. Significant hub and hospital level variation were observed in platelet transfusion incidence and use based on case mix indices. The median (IQR) storage duration at the time of transfusion was 4.5 (3.7-5.1) days, with 15.3% of platelet units stored more than 5 days. Of the apheresis products, 100% were single-donor and leukoreduced, 59.7% were gamma irradiated, and 40.3% underwent PR. A total of 65.6% of PR platelets were stored in PAS. One-third of platelet components (33.5%) were from female donors, and 21.8% were from donors younger than 40 years of age.

### Pretransfusion Platelet Counts, Transfusion Dosing, and Platelet Count Increments

In neonates, 32.2% of platelet transfusions were given at pretransfusion platelet counts lower than 25 × 10^3^/µL, the remaining were given at higher pretransfusion platelet counts (including 38.5% at >25-50 × 10^3^/µL, 19.6% at >50-100 × 10^3^/µL, and 9.7% at >100 × 10^3^/µL) (Table 2; eFigure 2 in Supplement 1). Among older children, 26 570 transfusion events were analyzed after the aforementioned exclusions. Of these events, 19.0% of platelet transfusions were given at pretransfusion platelet counts lower than 10 × 10^3^/µL and the remaining were at higher pretransfusion platelet counts (including 3.0% at >100 × 10^3^/µL). The median transfusion dose was 14.9 mL/kg in neonates and 9.6 mL/kg in older children (dose analysis restricted to children <20 kg, as those with weights >20 kg likely received 1 full unit of platelets and not a weight-based dose).

**Table 2.** Median (IQR) of 24-Hour Platelet Count Measures Before and After Transfusion, and Platelet Increment After Transfusion for Platelet Transfusions, Stratified by Pretransfusion Counts, for Neonatal (≤28 Days of Age) and Older Pediatric Patient (>28 Days to <18 Years) Encounters

Pretreatment platelet count, × 10^3^/µL	No. (%)	Median (IQR)				
		Time from pretransfusion to transfusion, h	Platelet count, × 10^3^/µL		Time from transfusion to posttransfusion, h	Posttransfusion platelet increment, × 10^3^/µL
			Pretreatment	Posttreatment		
Neonatal encounters						
<25	209 (32.2)	5.4 (2.6 to 6.7)	16 (11 to 20)	36 (18 to 64)	6.1 (3.6 to 9.3)	21 (5 to 45)
>25-50	250 (38.5)	4.3 (2.6 to 7.1)	35 (29 to 42)	63 (45 to 94)	7.4 (4.1 to 12.6)	28 (7 to 62)
>50-100	127 (19.6)	4.7 (2.1 to 7.2)	63 (54 to 76)	109 (68 to 136)	6.4 (3.7 to 12.4)	37 (7 to 68)
>100	63 (9.7)	1.7 (1.1 to 4.4)	162 (115 to 255)	156 (108 to 237)	5 (2.2 to 8.1)	−24 (−49 to 8)
Total	649 (100)	4.4 (2.2 to 6.9)	34 (20 to 54)	65 (39 to 115)	6.4 (3.6 to 10.6)	25 (2 to 55)
Older pediatric encounters						
≤10	5038 (19.0)	5.5 (2.8 to 7.1)	7 (5 to 8)	22 (11 to 39)	15.1 (6.3 to 19.2)	15 (2 to 32)
>10-20	7045 (26.5)	5.8 (2.9 to 8.4)	14 (12 to 17)	30 (19 to 48)	12.1 (5.3 to 18.4)	15 (5 to 34)
>20-50	10 347 (38.9)	5.5 (2.6 to 7.9)	30 (25 to 39)	47 (32 to 67)	9.5 (4.1 to 17.2)	14 (2 to 33)
>50-100	3351 (12.6)	2.9 (1.7 to 6.2)	65 (56 to 78)	72 (53 to 100)	7.7 (3.6 to 14.4)	5 (−14 to 31)
>100	789 (3.0)	2.1 (1.2 to 3.2)	134 (113 to 180)	119 (88 to 172)	6.8 (3 to 14)	−22 (−48 to 3)
Total	26 570 (100)	5.2 (2.4 to 7.6)	22 (11 to 40)	41 (24 to 66)	10.6 (4.5 to 17.9)	13 (2 to 32)

To convert platelet count to SI units ( × 10^9^/L), multiply by 1.0.

Neonates had significantly higher median (IQR) pretransfusion platelet counts compared with older pediatric patients (34 × 10^3^/µL [20-54 × 10^3^/µL] vs 22 × 10^3^/µL [11-40 × 10^3^/µL]; *P* < .001) (Table 2). In neonates, posttransfusion PI was highest for pretransfusion platelet counts lower than 25 × 10^3^/µL (median, 21 × 10^3^/µL [IQR, 5-45 × 10^3^/µL]) and decreased for higher counts, becoming negative (median, −25 × 10^3^/µL [IQR, −49 to 8 × 10^3^/µL]) for counts higher than 100 × 10^3^/µL (Table 2; eFigure 2 in Supplement 1). Similarly, among infants and older children, PIs were highest for counts lower than 10 × 10^3^/µL (median, 15 × 10^3^/µL [IQR, 2-32 × 10^3^/µL]) and decreased for higher counts, becoming negative (median, −22 × 10^3^/µL [IQR, −48 to 3 × 10^3^/µL]) for counts higher than 100 × 10^3^/µL.

### Platelet Processing Steps, Donor Factors, and Associations With Platelet Count Increments

Platelet components stored in PAS had reduced odds of posttransfusion PI higher than 15 × 10^3^/µL compared with platelet components not stored in PAS (adjusted OR [AOR], 0.32 [95% CI, 0.27-0.37]) (Figure; eTable 3 in Supplement 1). Platelet storage (longer than 3 days) was associated with decreased odds of PI higher than 15 × 10^3^/µL, with AORs ranging from 0.67 (95% CI, 0.58-0.76) to 0.82 (95% CI, 0.76-0.88). Pathogen-reduced platelet components were associated with lower odds of achieving an increment higher than 15 × 10^3^/µL (AOR, 0.82 [95% CI, 0.73-0.92]).

**Figure.**
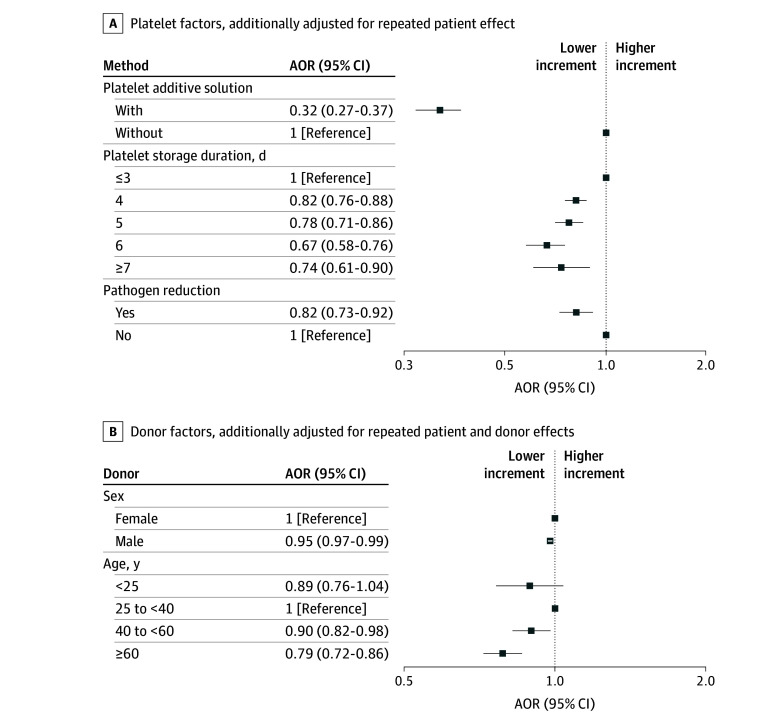
Adjusted Odds Ratios (AORs) and 95% CIs of Donor and Platelet Factors Associated With Platelet Increment Higher Than 15 × 10^3^/µL Squares represent AORs; lines, 95% CIs. To convert platelet count to SI units ( × 10^9^/L), multiply by 1.0.

Evaluating the various donor factors assessing PI, male sex was associated with lower odds (AOR, 0.92 [95% CI, 0.86-0.98]) of posttransfusion PI higher than 15 × 10^3^/µL per transfusion event (Figure; eTable 3 in Supplement 1). Donor age of 40 years or older compared with donor age 25 to less than 40 years was independently associated with lower odds of a PI higher than 15 × 10^3^/µL platelet increment (AOR, 0.79 [95% CI, 0.72-0.86]).

### Donor and Platelet Processing Factors and Association With Overall Platelet Transfusion Burden

Several platelet factors were associated with higher overall platelet transfusion burden. Use of PR platelets (adjusted rate ratio [ARR], 1.05 [95% CI, 1.02-1.07]), platelets in PAS (ARR, 1.44 [95% CI, 1.40-1.47]), storage duration longer than 3 days (ARR, 1.11 [95% CI, 1.09-1.13] for 4 to <5 days and ARR, 1.28 [95% CI, 1.26-1.30] for ≥5 days), and donor age of 40 years or older (ARR, 1.15 [95% CI, 1.13-1.17] for 40 to <60 years and ARR, 1.10 [95% CI, 1.08-1.12] for ≥60 years) on the first transfusion were associated with a significantly higher rate of receiving a subsequent transfusion (Table 3).

**Table 3.** Association of Donor and Platelet Factors With Transfusion Burden Expressed as ARRs of Overall Platelet Transfusions Per Encounter

Platelet characteristics at first transfusion	No. of encounters	RR (95% CI)^a^	ARR (95% CI)^a^
Model 1			
Pathogen reduction			
No	2946	1 [Reference]	1 [Reference]
Yes	1788	1.22 (1.20-1.24)	1.05 (1.02-1.07)
Platelet additive solution			
No	3571	1 [Reference]	1 [Reference]
Yes	1163	1.36 (1.34-1.38)	1.44 (1.40-1.47)
Platelet storage duration, d			
<4	1499	1 [Reference]	1 [Reference]
4 to <5	1659	1.11 (1.09-1.12)	1.11 (1.09-1.13)
≥5	1417	1.13 (1.11-1.15)	1.28 (1.26-1.30)
Observations used in adjusted models	4575		
Model 2			
Donor sex			
Male	3001	0.99 (0.98-1.01)	0.98 (0.97-0.99)
Female	1564	1 [Reference]	1 [Reference]
Unspecified	169	1.26 (1.21-1.30)	NA
Donor age, y			
<40	977	1 [Reference]	1 [Reference]
40 to <60	1633	1.14 (1.12-1.16)	1.15 (1.13-1.17)
≥60	1778	1.10 (1.08-1.12)	1.10 (1.08-1.12)
Observations used in adjusted models	4388		

Abbreviations: ARR, adjusted rate ratio; NA, not applicable; RR, rate ratio.

^a^ ARR includes an offset of log (length of hospital stay).

### Donor and Platelet Component Characteristics and Associations With Clinical Outcomes

In multivariable analysis, no donor or platelet characteristics were associated with a difference in risk of hospital discharge after 7 days (eg, ARR, 1.03 [95% CI, 0.89-1.20] for PR) (Table 4). Further, the rate of mortality was unchanged among platelet characteristics after an offset for the hospital length of stay of the encounter (eg, ARR, 0.66 [95% CI, 0.42-1.04] for PR).

**Table 4.** Univariate and Multivariable Analysis of Platelet and Donor Characteristics and Clinical Outcomes in Patients at the First Transfusion

Platelet characteristics at first transfusion	No. of encounters	Hospital length of stay or discharge in 7 d		Mortality^a^	
		RR (95% CI)	ARR (95% CI)	RR (95% CI)	ARR (95% CI)
Model 1 (n = 4575)					
Pathogen reduction					
No	2946	1 [Reference]	1 [Reference]	1 [Reference]	1 [Reference]
Yes	1788	1.07 (0.96-1.20)	1.03 (0.89-1.20)	0.83 (0.63-1.10)	0.66 (0.42-1.04)
Platelet additive solution					
No	3571	1 [Reference]	1 [Reference]	1 [Reference]	1 [Reference]
Yes	1163	1.10 (0.97-1.25)	1.11 (0.94-1.31)	1.00 (0.74-1.36)	1.49 (0.92-2.42)
Platelet storage duration, d					
<4	1499	1 [Reference]	1 [Reference]	1 [Reference]	1 [Reference]
4 to <5	1659	1.06 (0.95-1.18)	1.04 (0.94-1.17)	1.01 (0.73-1.38)	1.08 (0.78-1.49)
≥5	1417	1.08 (0.96-1.20)	1.11 (0.98-1.24)	1.13 (0.82-1.55)	1.11 (0.80-1.55)
Model 2 (n = 4387)					
Donor sex					
Male	3001	0.96 (0.88-1.06)	0.97 (0.89-1.07)	0.95 (0.72-1.24)	0.92 (0.70-1.22)
Female	1564	1 [Reference]	1 [Reference]	1 [Reference]	1 [Reference]
Not specified	169	0.93 (0.72-1.20)	NA	0.66 (0.27-1.62)	NA
Donor age, y					
<40	977	1 [Reference]	1 [Reference]	1 [Reference]	1 [Reference]
40 to <60	1633	1.01 (0.90-1.15)	1.01 (0.90-1.15)	1.08 (0.76-1.55)	1.09 (0.76-1.56)
≥60	1778	0.99 (0.88-1.12)	0.99 (0.88-1.12)	1.04 (0.73-1.48)	1.05 (0.74-1.50)

Abbreviations: ARR, adjusted rate ratio; NA, not applicable; RR, rate ratio.

^a^ Mortality adjusted for an offset of hospital length of stay.

## Discussion

This large, multicenter, donor-component-recipient–linked study including nearly 250 000 inpatient encounters of neonates and children assessed the epidemiology of platelet transfusions, donor characteristics, platelet processing steps associated with posttransfusion PIs, and overall transfusion burden and selected clinical outcomes.

In this study, 3.6% of patients overall received platelet transfusions. This percentage matches the prior reported platelet use rate of 3% to 4% in hospitalized children in prior analyses using REDS-III and the pediatric health information databases^7,21^

In our analyses, after excluding patients with bleeding, the median pretransfusion platelet counts as well as posttransfusion PIs in neonates were significantly higher than in older children. This may be explained by a higher median platelet transfusion dose in neonates (14.9 mL/kg) than in older children (9.6 mL/kg). However, there may also be different disease processes that explain this difference.

Platelet transfusion thresholds remain a topic of ongoing debate. International clinical practice platelet transfusion guidelines were recently released.^22^ For consumptive thrombocytopenia in neonates without major bleeding, when a platelet count is less than 25 × 10^3^/µL, platelet transfusion is a strong recommendation with a high certainty of evidence.^1^ Although not directly applicable to our population (since infants with <2500 g birth weight were excluded), the PLANET-2 study randomized preterm neonates less than 34 weeks’ gestation (<1500 g) to platelet thresholds of 25 × 10^3^/µL vs 50 × 10^3^/µL for prophylactic platelet transfusions. Infants in higher threshold groups had a significantly higher rate of death or major bleeding than the lower threshold group.^14^ Our analyses did not include preterm neonates; however, after excluding assumed bleeding, platelet transfusions were given at pretransfusion platelet counts lower than 25 × 10^3^/µL in only one-third (32.2%) of neonates, with about one-third (38.5%) receiving platelets at the 25 to 50 × 10^3^/µL range, and about one-third (29.3%) at higher than 50 × 10^3^/µL pretransfusion platelet counts. In more than 80% of older children, transfusions were given with higher than 10 × 10^3^/µL pretransfusion counts. This underscores the substantial variability in platelet transfusion practices, even among neonates, highlighting the need to tailor evidence-based guidelines to different patient populations.

For older children, PIs were higher at low pretransfusion platelet counts, and negative increments were observed at pretransfusion counts higher than 100 × 10^3^/µL for all age groups. Underlying comorbidities, such as fever, sepsis, and splenomegaly, and immune responses (eg, HLA antibodies), often result in lower PI due to increased platelet consumption or destruction.^23^ The PACER trial found clinical factors including sepsis, disseminated intravascular coagulation, bone marrow suppression, and irradiation as risk factors for poor PI^24^ In addition, while we a posteriori excluded patients with bleeding, these results could also be artifactual based on timing of posttransfusion platelet counts, especially in multiply transfused patients. These results highlight not only the clinical complexity of transfusion responses but also the potential for bias introduced by a posteriori exclusion of patients with bleeding, warranting careful interpretation of these findings.

In our analysis, female donors and donor ages younger than 40 years were independently associated with higher posttransfusion PI than donors 40 to less than 60 years and those 60 years or older. Some of these results could be explained by metabolomics analyses, in which in platelets from older donors and male donors, higher levels of metabolites, such as Krebs cycle and pentose phosphate pathway intermediates and byproducts, deaminated purines, and long-chain fatty acids, were associated with poor posttransfusion recoveries.^8^

In our study, significantly lower PIs were observed for platelets in PAS, PR, and stored longer than 3 days. Post hoc analyses of the Trial to Reduce Alloimmunization to Platelets study showed that the platelet factors associated with improved platelet responses were transfusing ABO-compatible platelets or platelets stored for 48 hours or less.^23^ Other studies noted lower PI with prolonged storage and PAS use.^25,26^

These donor and platelet factors not only are associated with PI but also with the overall transfusion burden. Despite these associations, these factors were not associated with the clinical outcomes, including the overall length of hospital stay or mortality rate. These results have future implications for precision transfusion medicine efforts, with the goal for the best donor-component-recipient match aiming for the highest transfusion effectiveness while minimizing transfusion burden and overall transfusion-related adverse events. It also underscores the importance of optimizing the transfusion decision based on patients’ overall clinical picture rather than aiming for a specific transfusion increment.

### Limitations

This study has limitations. By design, REDS IV-P is a retrospective study of transfusion practices. Therefore, we were unable to establish temporal correlations of many parameters (eg, pretransfusion laboratory values and location) with clinical outcomes. *International Statistical Classification of Diseases, Tenth Revision* diagnostic codes were abstracted at the time of discharge. The exact transfusion indication, whether for prophylactic or therapeutic purposes, could not be determined from the database, and any interpretations of the use or variability based on these data will be speculative. It is unclear whether these transfusions were performed preprocedure or before or after surgery or given to treat a hemostatic abnormality or a platelet function defect, or whether the therapy was guided by viscoelastic testing or a laboratory-based platelet number. The issued transfusion volumes were used as a surrogate for the transfused volume where such results were missing from the database, although the actual transfused volume could be different from the issued volume. Also, there was approximation by counting any aliquot or pediatric dose as a single transfusion event, although volumes were calculated as milliliters per kilogram. The exact platelet concentration within each platelet unit was unknown; thus, the association of dose with PI could not be assessed but would be expected to have met the minimum dose per the regulatory requirements. Although an analysis of between-center variability influencing transfusion practices would have been informative, this information is not included in the REDS-IV-P Vein-to-Vein database. The bleeding definition was developed post hoc by the study coauthors, is inherently restricted by the variables available in the REDS-IV-P database, and needs prospective and external validation. We also could not account for other immune and nonimmune factors affecting PI. In our study, platelet factors were not associated with higher odds of longer length of hospital stay or mortality rate. Although we adjusted for the number of transfusions, we could not adjust for the underlying severity of illness. These limitations underscore the importance of cautious interpretation, as reliance on administrative data makes it difficult to disentangle the impact of transfusions from underlying illness severity or establish causal relationships.^4,27^

## Conclusions

In this multicenter retrospective donor-component-recipient–linked cohort study of 249 340 inpatient encounters in neonates and children, platelet transfusions were given to 3.6% of patients overall. Use of PAS, PR, longer stored units, or donor age older than 40 years was associated with lower posttransfusion PI as well as higher overall platelet transfusion burden. These factors were not associated with the overall length of hospital stay or mortality rate. These findings underscore the importance of precision transfusion medicine applications aiming for the best combinations of factors for optimizing transfusion outcomes. These results have important implications for transfusion practices and outcomes for platelet transfusion in neonates and children but need validation in well-designed prospective studies.

## Notes

Supplement 1. **eFigure 1.** Flowchart showing total platelet transfusion events, encounters, and patients **eFigure 2.** Density plot showing distributions of post-transfusion platelet increment in neonates and older children, by pre-transfusion platelet count categories after excluding bleeding patients **eTable 1.** Comparisons between the subjects included and excluded from study population and percentage of missing variables **eTable 2.** Platelet component characteristics and donor factors for platelet transfusion episodes **eTable 3.** Platelet and donor-specific factors associated with unadjusted and adjusted odds of platelet increment above 15K **eAppendix.** Bleeding definition per REDS-IV-P working group

Supplement 2.
Data Sharing Statement
