# Supplementary material for: Platelet Transfusion Practices and Outcomes in Neonates and Children
Source: JAMA Netw Open. 2026 Jan 28;9(1):e2554531. doi: 10.1001/jamanetworkopen.2025.54531 (PMC12853206; doi:10.1001/jamanetworkopen.2025.54531)
Supplement: Supplement 1. — eFigure 1. Flowchart showing total platelet transfusion events, encounters, and patients eFigure 2. Density plot showing distributions of post-transfusion platelet increment in neonates and older children, by pre-transfusion platelet count categories after excluding bleeding patients eTable 1. Comparisons between the subjects included and excluded from study population and percentage of missing variables eTable 2. Platelet component characteristics and donor factors for platelet transfusion episodes eTable 3. Platelet and donor-specific factors associated with unadjusted and adjusted odds of platelet increment above 15K eAppendix. Bleeding definition per REDS-IV-P working group [file jamanetwopen-e2554531-s001.pdf]

# Supplementary Online Content

Goel R, Karam O, Warden DE, et al. Platelet transfusion practices and outcomes in term neonates and children. *JAMA Netw Open*. 2026;9(1):e2554531.
doi:10.1001/jamanetworkopen.2025.54531

**eFigure 1.** Flowchart showing total platelet transfusion events, encounters, and patients

**eFigure 2.** Density plot showing distributions of post-transfusion platelet increment in neonates and older children, by pre-transfusion platelet count categories after excluding bleeding patients

**eTable 1.** Comparisons between the subjects included and excluded from study population and percentage of missing variables

**eTable 2.** Platelet component characteristics and donor factors for platelet transfusion episodes

**eTable 3.** Platelet and donor-specific factors associated with unadjusted and adjusted odds of platelet increment above 15K

**eAppendix.** Bleeding definition per REDS-IV-P working group

This supplementary material has been provided by the authors to give readers additional information about their work.

© 2026 Goel R et al. *JAMA Network Open*.

**eFigure 1. Flowchart showing total platelet transfusion events, encounters, and patients** as analyzed for assessment of post-transfusion platelet increments stratified for neonates (<28 days and >2500gm) and older pediatric patients (>28 days and >2500gms) after excluding patients with assumed bleeding as defined per methods, patients on ECMO, and patients on cardio-pulmonary bypass. Note that neonatal patients may have encounters that last longer than 28 days, thus the same patients and encounters can be in both the neonatal and older pediatric patient sub-populations.

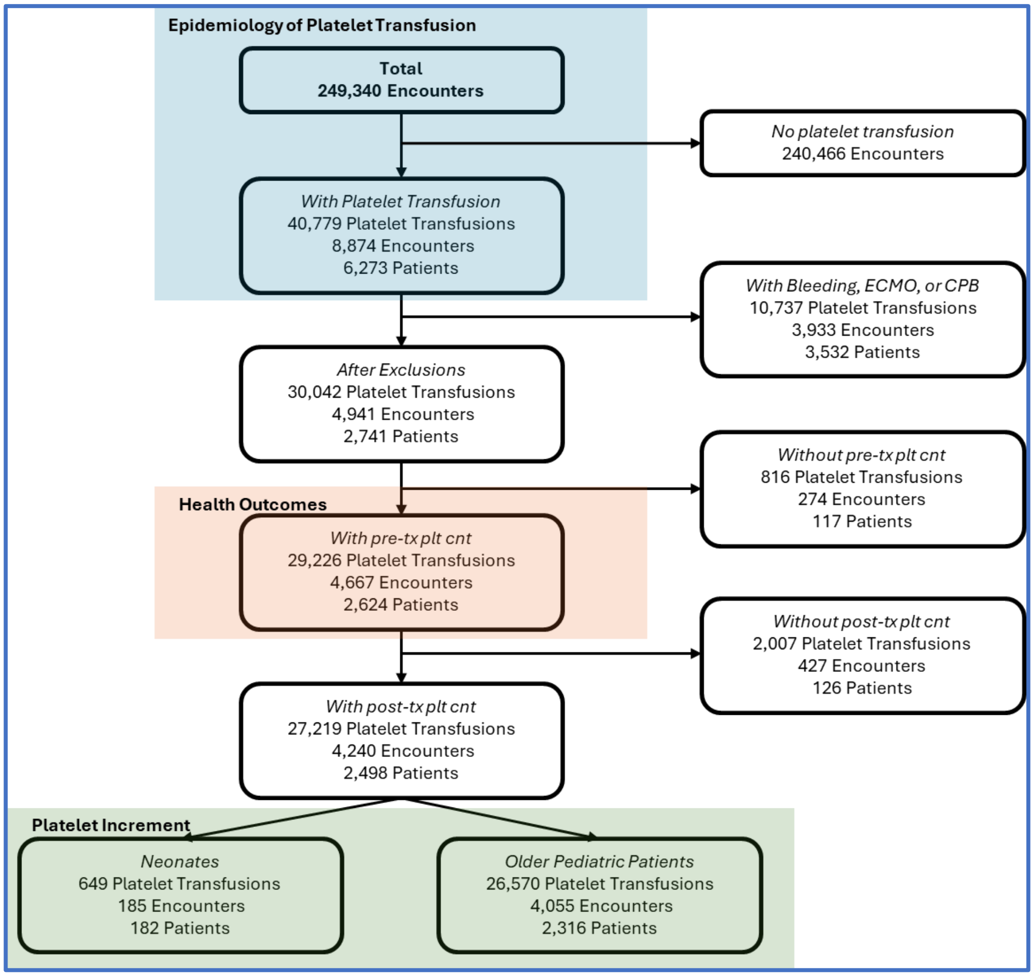


© 2026 Goel R et al. *JAMA Network Open*.

**eFigure 2**. **Density plot showing distributions of post-transfusion platelet increment in neonates and older children, by pre-transfusion platelet count categories after excluding bleeding patients** (bleeding as defined by REDS-IV-P working group)

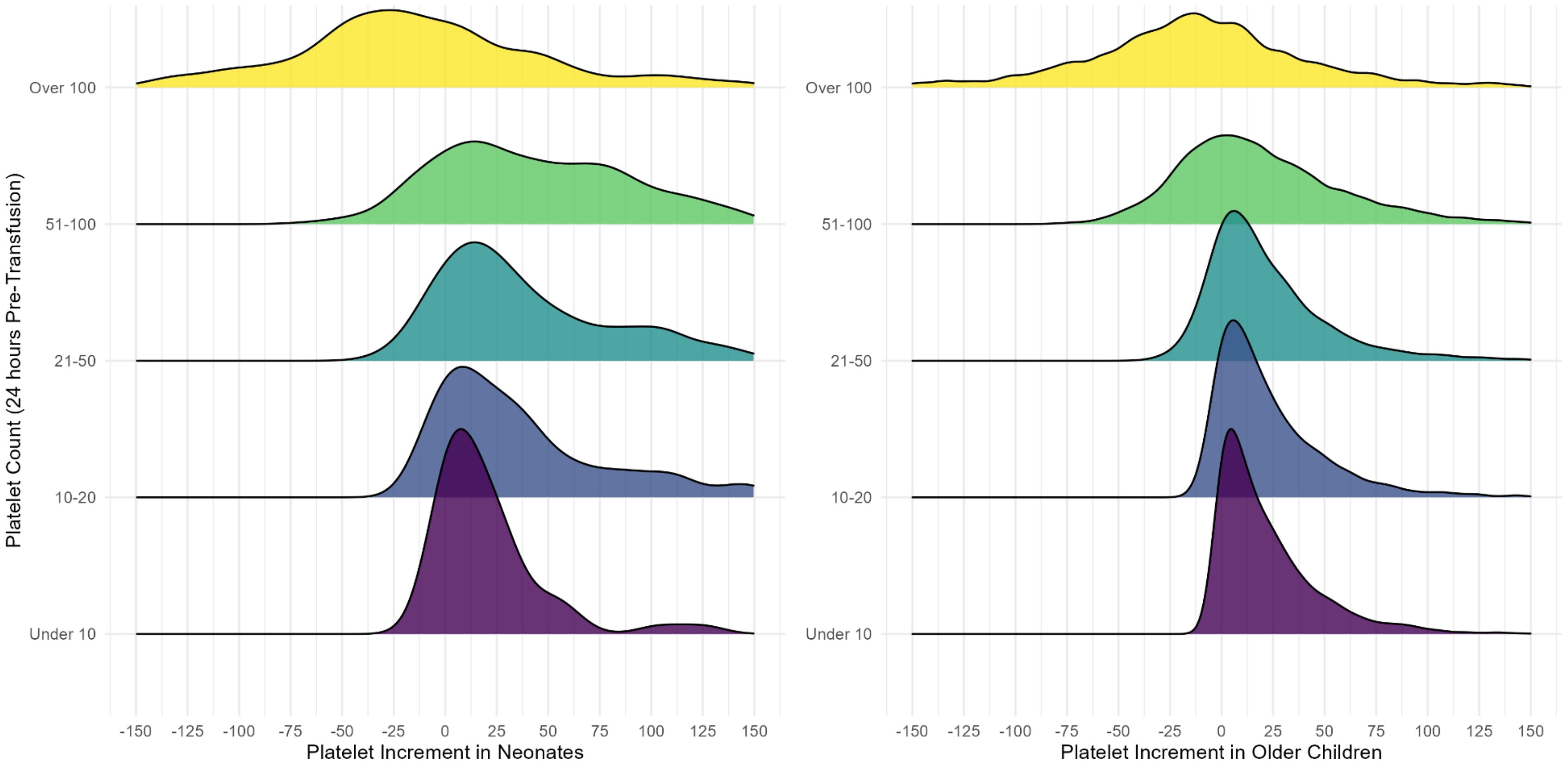


© 2026 Goel R et al. *JAMA Network Open*.

## eTable 1. Comparisons between the subjects included and excluded from study population and percentage of missing variables

| | Total Inpatient Transfusions | | Excluded in All Analyses | | | | Before Model Specific Exclusions | | Increment Models | | Clinical Outcomes and Transfusion Burden Models | | | |
|---|---|---|---|---|---|---|---|---|---|---|---|---|---|---|
| Transfusion Characteristics | Total (N=40,779)[a] | | Excluded due to Bleeding/ECMO/CPB | | Exlcuded due to no pre-tx plt cnt | | After Exclusions (N=29,226) | | Exlcuded due to no post-tx plt cnt[b] | | Excluded due to missing component factors[ce] | | Excluded due to missing donor factors[de] | |
| | N | % | N | Excluded % | N | Excluded % | N | % | N | Excluded % | N | Missing % | N | Missing % |
| **Total platelet transfusions** | 40,779 | 100.0% | 10,797 | 26.5% | 2,314 | 5.7% | **29,226** | **100.00%** | 2,007 | 6.9% | 1,335 | 4.6% | 2,519 | 8.6% |
| **Sex** | | | N | Col. % | N | Col. % | | | N | Col. % | N | Col. % | N | Col. % |
| Male | 22,578 | 55.4% | 6,007 | 55.6% | 1,324 | 57.2% | 16,105 | 55.11% | 1,109 | 55.3% | 777 | 58.2% | 1,454 | 57.7% |
| Female | 18,200 | 44.6% | 4,729 | 43.8% | 990 | 42.8% | 13,121 | 44.89% | 898 | 44.7% | 558 | 41.8% | 1,065 | 42.3% |
| Unknown | 1 | 0.0% | 1 | 0.0% | 0 | 0.0% | 0 | 0.00% | 0 | 0.0% | 0 | 0.0% | 0 | 0.0% |
| **Weight in kg (median (IQR))** | 25.9 | 11.69-52.99 | 9.35 | 3.99-25.59 | 17.64 | 6.69-48.49 | 36.09 | 15.44-56.79 | 30.79 | 16.09-53.89 | 30.79 | 15.09-47.99 | 31.79 | 14.99-54.49 |
| **Age (years) (median (IQR))** | 6.6 | 1.99-12.99 | 1.21 | 0.02-7.69 | 3.75 | 0.39-11.70 | 8.37 | 3.42-13.90 | 8.12 | 3.75-13.22 | 9.07 | 3.42-14.72 | 9.96 | 3.32-14.64 |
| **Age Group** | | | | | | | | | | | | | | |
| <1 years | 7,791 | 19.1% | 5,193 | 48.1% | 838 | 36.2% | 2,529 | 8.65% | 139 | 6.9% | 114 | 8.5% | 284 | 11.3% |
| 1 to <6 | 11,544 | 28.3% | 2,478 | 23.0% | 541 | 23.4% | 8,853 | 30.29% | 643 | 32.0% | 392 | 29.4% | 690 | 27.4% |
| 6 to <13 | 11,265 | 27.6% | 1,574 | 14.6% | 478 | 20.7% | 9,393 | 32.14% | 714 | 35.6% | 410 | 30.7% | 640 | 25.4% |
| 13 to < 18 | 10,179 | 25.0% | 1,492 | 13.8% | 457 | 19.7% | 8,451 | 28.92% | 511 | 25.5% | 419 | 31.4% | 905 | 35.9% |
| **Race** | | | | | | | | | | | | | | |
| Asian | 2,837 | 7.0% | 607 | 5.6% | 195 | 8.4% | 2,155 | 7.37% | 157 | 7.8% | 68 | 5.1% | 124 | 4.9% |
| Black | 4,503 | 11.0% | 1,113 | 10.3% | 243 | 10.5% | 3,303 | 11.30% | 190 | 9.5% | 126 | 9.4% | 380 | 15.1% |
| Multiple Races reported | 261 | 0.6% | 110 | 1.0% | 21 | 0.9% | 151 | 0.52% | 11 | 0.5% | 3 | 0.2% | 3 | 0.1% |
| Native American/American Indian | 321 | 0.8% | 97 | 0.9% | 7 | 0.3% | 218 | 0.75% | 11 | 0.5% | 2 | 0.1% | 7 | 0.3% |
| Native Hawaiian/Pacific Islander | 248 | 0.6% | 50 | 0.5% | 17 | 0.7% | 197 | 0.67% | 18 | 0.9% | 0 | 0.0% | 16 | 0.6% |
| Race reported as other | 6,544 | 16.0% | 1,529 | 14.2% | 436 | 18.8% | 4,892 | 16.74% | 347 | 17.3% | 275 | 20.6% | 304 | 12.1% |
| Unknown | 6,660 | 16.3% | 2,727 | 25.3% | 317 | 13.7% | 3,848 | 13.17% | 223 | 11.1% | 239 | 17.9% | 528 | 21.0% |
| White | 19,405 | 47.6% | 4,504 | 41.7% | 1,078 | 46.6% | 14,462 | 49.48% | 1,050 | 52.3% | 622 | 46.6% | 1,157 | 45.9% |
| **Pathogen reduction** | | | | | | | | | | | | | | |
| No | 24,671 | 60.5% | 6,902 | 63.9% | 1,130 | 48.8% | 17,249 | 59.02% | 1,279 | 63.7% | 720 | 53.9% | 946 | 37.6% |
| Yes | 16,108 | 39.5% | 3,835 | 35.5% | 1,184 | 51.2% | 11,977 | 40.98% | 728 | 36.3% | 615 | 46.1% | 1,573 | 62.4% |
| Platelet Additive Solution | | | | | | | | | | | | | | |
| No | 29,561 | 72.5% | 8,313 | 77.0% | 1,605 | 69.4% | 20,639 | 70.62% | 1,488 | 74.1% | 579 | 43.4% | 674 | 26.8% |
| Yes | 11,218 | 27.5% | 2,424 | 22.5% | 709 | 30.6% | 8,587 | 29.38% | 519 | 25.9% | 756 | 56.6% | 1,845 | 73.2% |
| **Platelet Storage Duration** | | | | | | | | | | | | | | |
| <4 days | 13,910 | 34.1% | 3,312 | 30.7% | 963 | 41.6% | 10,232 | 35.01% | 636 | 31.7% | 0 | 0.0% | 334 | 13.3% |
| 4-5 days | 13,237 | 32.5% | 3,124 | 28.9% | 702 | 30.3% | 9,865 | 33.75% | 702 | 35.0% | 0 | 0.0% | 599 | 23.8% |
| 5+ days | 11,648 | 28.6% | 3,673 | 34.0% | 529 | 22.9% | 7,794 | 26.67% | 589 | 29.3% | 0 | 0.0% | 251 | 10.0% |

© 2026 Goel R et al. *JAMA Network Open*.

| | | | | | | | | | | | | | | |
|---|---|---|---|---|---|---|---|---|---|---|---|---|---|---|
| Missing | 1,984 | 4.9% | 628 | 5.8% | 120 | 5.2% | 1,335 | 4.57% | 80 | 4.0% | 1,335 | 100.0% | 1,335 | 53.0% |
| **Donor Sex** | | | | | | | | | | | | | | |
| Male | 25,072 | 61.5% | 6,470 | 59.9% | 1,434 | 62.0% | 18,082 | 61.87% | 1,278 | 63.7% | 0 | 0.0% | 762 | 30.3% |
| Female | 13,643 | 33.5% | 3,623 | 33.6% | 755 | 32.6% | 9,749 | 33.36% | 644 | 32.1% | 0 | 0.0% | 363 | 14.4% |
| Unspecified | 2,064 | 5.1% | 644 | 6.0% | 125 | 5.4% | 1,395 | 4.77% | 85 | 4.2% | 1,335 | 100.0% | 1,394 | 55.3% |
| **Donor Age in years** | | | | | | | | | | | | | | |
| <40 | 8,881 | 21.8% | 2,459 | 22.8% | 453 | 19.6% | 6,241 | 21.35% | 444 | 22.1% | 0 | 0.0% | 0 | 0.0% |
| 40 to <60 | 13,622 | 33.4% | 3,559 | 33.0% | 771 | 33.3% | 9,791 | 33.50% | 691 | 34.4% | 0 | 0.0% | 0 | 0.0% |
| ≥60 | 14,772 | 36.2% | 3,780 | 35.0% | 891 | 38.5% | 10,675 | 36.53% | 730 | 36.4% | 0 | 0.0% | 0 | 0.0% |
| Missing | 3,504 | 8.6% | 939 | 8.7% | 199 | 8.6% | 2,519 | 8.62% | 142 | 7.1% | 1,335 | 100.0% | 2,519 | 100.0% |

[a]Study population used for describing the epidemiology of pediatric platelet transfusions (Table 1)

[b]Excluded in analyses investigating platelet increment (Supplemental Table 2, Supplemental Table 1, and Supplemental figure 2)

[c]Excluded in modeling of component factors on transfusion burden, platelet increment, and health outcomes (Figure 1, Tables 3, and 4). Data missing due to blood products fron non-REDS blood centers.

[d]Excluded in modeling of donor factors on transfusion burden, platelet increment, and health outcomes (Figure 1, Tables 3, and 4). Data missing due to blood products fron non-REDS blood centers.

[e]Modeling of donor and component factors was performed on a per encounter basis. Characteristics of transfusions within an encounter were combined to repsent the whole encounter. These exclusions do not inherently remove specific patients.

© 2026 Goel R et al. *JAMA Network Open*.

**eTable 2. Platelet component characteristics and donor factors for platelet transfusion episodes**

| Encounter characteristic | Platelet transfusion episodes among 8874 encounters with at least 1 platelet transfusion, No. (%) |
|---|---|
| Total platelet transfusion episodes | 40 779 (100) |
| No. of platelet transfusions per patient encounter, median (IQR) | 2 (1-4) |
| Platelet collection method | |
| Whole blood-derived | 0 |
| Apheresis derived | 40 779 (100) |
| Concomitant receipt of red blood cells[a] | 10 549 (25.9) |
| Concomitant receipt of plasma[a] | 4757 (11.7) |
| Concomitant receipt of cryoprecipitate[a] | 1311 (3.2) |
| Storage duration on day of transfusion, median (IQR), d | 4.5 (3.7-5.1) |
| Storage duration, d | |
| ≤3 | 7860 (19.3) |
| 4 | 11 534 (28.3) |
| 5 | 13 147 (32.2) |
| 6 | 3930 (9.6) |
| 7 | 2215 (5.4) |
| >7 | 109 (0.3) |
| Missing | 1984 (4.9) |
| Pathogen reduction[b] | |
| Yes | 16 108 (39.5) |
| No | 24 671 (60.5) |
| Platelet additive solution | |
| Yes | 11 080 (27.2) |
| No | 29 699 (72.8) |
| Platelet irradiation | |
| Yes | 24 365 (59.7) |
| No | 16 414 (40.3) |
| Donor sex | |
| Female | 13 643 (33.5) |
| Male | 25 072 (61.5) |

© 2026 Goel R et al. *JAMA Network Open*.

| | |
|---|---|
| Not specified | 2064 (5.1) |
| Donor age, y | |
| <25 | 1708 (4.2) |
| 25 to <40 | 7173 (17.6) |
| 40 to <60 | 13 622 (33.4) |
| ≥60 | 14 772 (36.2) |
| Unknown | 3504 (8.6) |
| Donor race | |
| Asian | 1025 (2.5) |
| Black | 329 (0.8) |
| White | 13 394 (32.8) |
| Other | 1032 (2.5) |
| Unknown[c] | 24 999 (61.3) |
| Donor ethnicity | |
| Hispanic or Latino | 1326 (3.3) |
| Not Hispanic or Latino | 14 220 (34.9) |
| Unknown or missing ethnicity[c] | 25 233 (61.9) |
| Donor ABO status | |
| O | 13 139 (32.2) |
| A | 16 857 (41.3) |
| B | 5432 (13.3) |
| AB | 5237 (12.8) |
| ABO mismatch status | |
| Match | 14 903 (36.5) |
| Minor mismatch | 6319 (15.5) |
| Major mismatch | 9758 (23.9) |
| Bidirectional | 884 (2.2) |
| Unknown | 8915 (21.9) |

[a]Concomitant transfusion was defined as being within 6 hours of a platelet transfusion.

[b]In total, 65% of pathogen-reduced platelets (n = 10 146) were stored in platelet additive solution.

[c]Donor race and ethnicity were unknown for most patients, as they were not reported originally. This variable was not included in model-building for association of donor platelet count increment or transfusion burden.

© 2026 Goel R et al. *JAMA Network Open*.

**eTable 3. Platelet and donor-specific factors associated with unadjusted and adjusted odds of platelet increment above 15K**

| | No. Patients | No. Platelet Transfusion Events | % with increment >15K | OR (95% CI) | aOR[a] (95% CI) |
|---|---|---|---|---|---|
| **Platelet transfusion episodes** | 2498 | 27219 | 48.3% | | |
| **MODEL 1: Association of Platelet Characteristics with Platelet Increment** | | | | | |
| **PAS** | | | | | |
| PAS + | 820 | 8,068 | 33.63% | **0.30 (0.26-0.34)** | **0.32 (0.27-0.37)** |
| PAS – (Ref) | 2,043 | 19,151 | 54.46% | 1 [Ref] | 1 [Ref] |
| **Platelet Storage Duration** | | | | | |
| ≤3 days (Ref) | 1,575 | 9,596 | 49.0% | 1 [Ref] | 1 [Ref] |
| 4 days | 1,700 | 9,163 | 47.2% | **0.84 (0.78-0.90)** | **0.82 (0.76-0.88)** |
| 5 days | 1,257 | 4,657 | 51.2% | **0.89 (0.81-0.97)** | **0.78 (0.71-0.86)** |
| 6 days | 551 | 1,885 | 51.0% | **0.81 (0.71-0.92)** | **0.67 (0.58-0.76)** |
| 7+ days | 316 | 663 | 55.8% | 0.98 (0.80-1.18) | **0.74 (0.61-0.90)** |
| **Pathogen Reduced** | | | | | |
| PR+ | 1,158 | 11,249 | 40.80% | **0.59 (0.54-0.65)** | **0.82 (0.73-0.92)** |
| PR- (Ref) | 1,831 | 15,970 | 53.56% | 1 [Ref] | 1 [Ref] |
| **MODEL 2: Association between Donor Characteristics with Platelet Increment** | | | | | |
| **Donor Sex** | | | | | |
| Male (Ref) | 2,173 | 16,804 | 48.3% | **0.92 (0.86-0.98)** | **0.92 (0.86-0.98)** |
| Female | 1,750 | 9,105 | 50.5% | 1 [Ref] | 1 [Ref] |
| Not specified | 437 | 1,310 | 33.3% | N/A | N/A |
| **Donor Age in years** | | | | | |
| <25 | 582 | 1,136 | 49.9% | 0.90 (0.77-1.05) | 0.89 (0.76-1.04) |
| 25 to <40 (Ref) | 1,309 | 4,661 | 52.5% | 1 [Ref] | 1 [Ref] |
| 40 to <60 | 1,756 | 9,100 | 50.5% | **0.92 (0.84-0.99)** | **0.90 (0.83-0.98)** |
| ≥60 | 1,786 | 9,945 | 47.8% | **0.83 (0.76-0.91)** | **0.79 (0.72-0.86)** |

Abbreviations: OR, odds ratio; aOR, adjusted Odds Ratio; CI, confidence interval; Ref=reference, PAS=Platelet Additive Solution, PR=Pathogen Reduced, IR=Irradiated.

[a]aOR=Adjusted Odds ratio; platelet characteristic models adjusted repeated patient effects; donor characteristics model adjusted for repeated patient effects, previous number of platelet donations from the donor median (IQR)=4(1-7), and length of time (months) since last platelet donation median (IQR) 25-(14-50).

© 2026 Goel R et al. *JAMA Network Open*.

**eAppendix. Bleeding definition per REDS-IV-P working group**: two of the following:

1. Co-transfusion of RBCs, Plasma, OR cryo, within 6 hours of platelet transfusion (before or after); OR
2. Drop in hemoglobin of 2 g/dL (absolute change, not percent of value, e.g. 10g/dL to 8g/dL) within 48 prior to platelet transfusion (both measures within 48 hours prior to transfusion, change using to closest to transfusion); OR
3. Drop in hematocrit of 6% (absolute change, not percent of value, e.g., 46% to 40%) within 48 prior to platelet transfusion (both measures within 48 hours prior to tx, change using to closest to transfusions); OR
4. Taking at least one of the following meds within 6 hours of platelet transfusions (before or after): 3-factor Prothrombin Complex Concentrate (PCC), 4-factor PCC, 4-factor activated PCC Activated factor VIIa, Andexanet alfa, Fibrinogen concentrate, Idarucizumab

And **no** hemolysis, defined as:

≤28 days old (NEONATES): Bilirubin > 20 at any time during encounter AND reticulocyte count >10% at any time during encounter

>28 days old (NON-NEONATES): TWO of the three criteria: LDH >1000 AND/OR Bilirubin > 2 AND/OR Haptoglobin <40mg/dL (or undetectable haptoglobin levels), at any time du

ring encounter.

© 2026 Goel R et al. *JAMA Network Open*.
