# Supplementary material for: Platelet Transfusion Practices and Outcomes in Neonates and Children
Source: JAMA Netw Open. 2026 Jan 28;9(1):e2554531. doi: 10.1001/jamanetworkopen.2025.54531 (PMC12853206; doi:10.1001/jamanetworkopen.2025.54531)
Supplement: Supplement 2. — Data Sharing Statement [file jamanetwopen-e2554531-s002.pdf]

Data Sharing Statement

Goel. Platelet Transfusion Practices and Outcomes in Neonates and Children. *JAMA Netw Open*. Published January 28, 2026. doi:10.1001/jamanetworkopen.2025.54531

Data
Data available: No
